# An Experimental Investigation on the Dynamic Response of Buried RC Pipes Induced by Falling Impact

**DOI:** 10.3390/s24030929

**Published:** 2024-01-31

**Authors:** Yingkang Yao, Nan Jiang, Guopeng Lyu, Jinshan Sun, Feng Yang

**Affiliations:** 1 State Key Laboratory of Precision Blasting, Jianghan University, Wuhan 430056, China; shanxiyao@sina.com (Y.Y.); sunjinshan@cug.edu.cn (J.S.); yangfeng@cug.edu.cn (F.Y.); 2 Hubei Key Laboratory of Blasting Engineering, Jianghan University, Wuhan 430056, China; 3 Faculty of Engineering, China University of Geosciences, Wuhan 430074, China

**Keywords:** falling impact, RC pipe, dynamic response, field experiment

## Abstract

Unexpected ground impacts can seriously affect the stability and operational safety of buried pipelines. In this paper, a full-scale modeling test of the dynamic response of a buried concrete pipeline under falling rock impact based on dynamic sensor testing was conducted. A commercially available reinforced concrete pipeline, buried in a clayey soil site, was used, and a 50 kg concrete ball was used to investigate the impact above the pipeline. Considering the purpose of the test, the falling process of the concrete ball and the surface vibration velocity induced by the touchdown of the concrete ball were monitored using a high-speed camera and a vibration signal tester, respectively. The dynamic response signals of the pipe under surface impact were tested using strain gauges and earth pressure gauges combined with dynamic sensors such as dynamic signal tester, and the dynamic response law was analyzed. The experimental results will provide a basis for the design of the impact resistance of reinforced concrete pipes.

## 1. Introduction

As a long-distance medium transport device, buried pipelines are widely distributed in various scenarios such as urban areas and mountainous regions, and they are subject to various disaster-causing risks from the ground.

In urban areas, demolition blasting techniques are commonly used for the demolition of old infrastructure and high-rise buildings. Demolition blasting can effectively solve the challenges faced in the demolition process, such as the narrow working space and the risk of adjacent buildings [1]. However, demolition blasting can also have many adverse effects on the surrounding environment. In the demolition process, the resulting collapsed material will exert a high kinetic energy impact on the ground, affecting the safety and stability of underground structures such as buried pipelines, tunnels, and so on [2,3]. In fact, in recent years, considering demolition and blasting projects that involve the collapsed material impacting the ground and subsequently affecting buried pipelines, the cracking phenomenon is not uncommon in subway tunnel lining.

In mountainous areas, “rockfall” disasters occur, which are similar to “demolition and collapse” disasters. Weathered and fissured rock on mountain slopes, once detached from the mountain under the action of many factors, can accumulate huge kinetic energy through a series of ways, such as rolling, jumping, falling, etc. [4], which threatens the safety of buildings and people below the slope. Long-distance buried pipelines passing through high mountain valleys face a higher risk of disaster caused by falling rocks [5].

Reinforced concrete pipelines are among the most common types of pipelines, widely used in the field of water supply and drainage. As a type of rigid pipeline, once a reinforced concrete pipeline is subjected to external influences, cracking damage may occur, and cracks easily expand and lead to the collapse of the pipeline, resulting in the inability to use the pipeline normally. To improve the resistance of buried pipelines to damage under the impact of “rockfall” or a “collapsing object”, it is first necessary to study the dynamic response of the pipeline considering the dynamic response law. Currently, many numerical simulation studies on the dynamic response characteristics and failure mechanisms of buried pipelines under impact are reported in the literature. For example, Liu [6] discussed the effects of pipe–soil relative stiffness, pipe burial depth, and impact energy on the stress distribution of buried pipes through numerical simulation tests. Tavakoli Mehrjardi [7] validated a numerical model based on an experimental study to assess the effect of the pipe burial depth, the drop height, the extent of the affected area, and soil damping on the response of buried pipes. Numerical simulation is recognized as an economical and efficient research method [8,9]; however, its reliability depends on accurate physical and mechanical parameters.

Carrying out experiments on buried pipelines impacted by falling rocks is still the most reliable approach to studying the dynamic response law of pipelines. Most of the existing literature surrounding rockfall impact tests on buried pipelines is based on the similarity principle, where the actual dimensions of the soil, rockfall, and pipeline are scaled down to the model size. Real-time monitoring sensors such as strain sensors, pressure sensors, etc., are utilized to capture and collect experimental data. This method is able to visualize the variation in the real parameters of the pipeline and provide theoretical as well as data support for research related to the dynamic response of buried pipelines impacted by falling rocks. For example, Anil et al. [10] conducted tests using drop hammers to impact buried pipelines to analyze the performance, strength, and energy absorption capacity of geofoam materials against impact forces. Feng et al. [11] comprehensively considered four basic parameters, namely, the rock mass, fall height, offset distance, and burial depth, to study the additional stresses generated by falling rocks’ impact on pipelines, and proposed a rapid method for assessing the additional stresses on pipelines. Obeid et al. [12] carried out a study on the mechanical response of composite pipes under dynamic impact by means of field tests and numerical simulations. All of the similar modeling experiments mentioned above have some assumptions and ignore some factors. In situ or prototype tests have higher reliability. For example, Yuqing et al. [13] conducted prototype tests on the dynamic response characteristics of buried pipes under the action of underground explosions and obtained meaningful data. At present, there are almost no studies in the literature focused on prototype testing to investigate the impact of falling objects on buried pipelines.

In this paper, a prototype test of a falling object impacting a buried reinforced concrete pipeline was designed and conducted. A commercially available reinforced concrete pipe was used for the study, and the pipe was buried in a clayey soil layer representative of soils in China. A variety of high-frequency real-time monitoring sensors were used in the experiment. The experimental results can provide a reliable basis for the study of the dynamic response characteristics of buried pipelines under impact and the prediction of the failure mechanism of pipelines under impact.

## 2. Materials and Methods

### 2.1. Experimental Program

#### 2.1.1. Selection of Experiment Site

**Figure 1:**
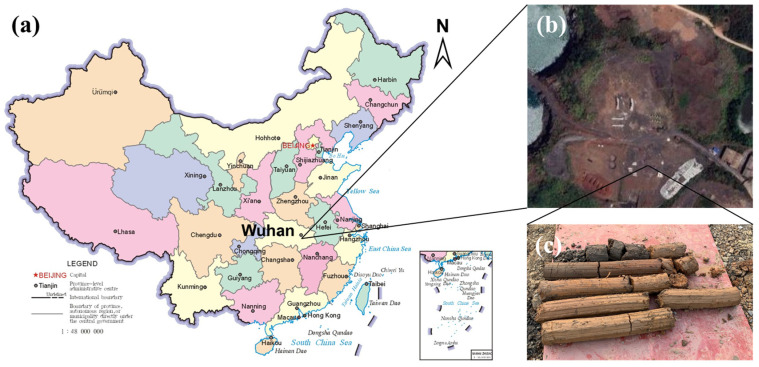
(**a**) Map of China; (**b**) experimental site in south of Wuhan; (**c**) clayey soil.

Clayey soils are widely distributed in the eastern, southern, and central regions of China. In the underground of the cities in these regions, there are a large number of water supply, drainage, gas, and other pipelines buried. The experiment site we selected is located in the south of Wuhan, Hubei Province, China, where the test area has a clayey soil layer more than 5 m thick (Figure 1). The physical and mechanical parameters of the clayey soil in this area are shown in Table 1.

#### 2.1.2. Selection of Experimental Pipes

**Figure 2:**
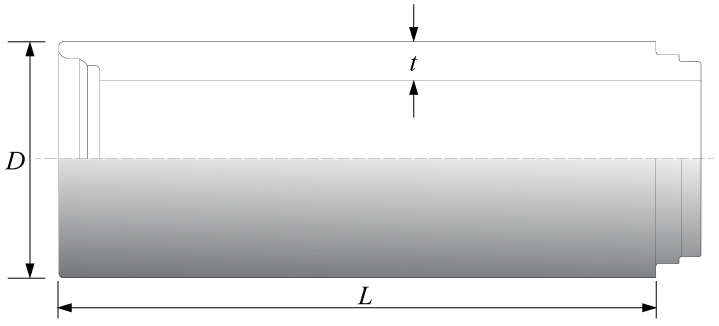
Tongue-and-groove concrete pipe.

In order to make the test universal and easy to operate, commercially available tongue-and-groove concrete pipes were selected for the test. The outer diameter *D* of the pipes was 1.8 m, the wall thickness *t* was 0.15 m, and the length *L* was 2 m. The piping system consisted of four sections of tongue-and-groove concrete pipes spliced together as the experimental pipeline. The specifications and strengths of the commercial reinforced concrete pipes are shown in Figure 2 and Table 2. The specifications of the pipes complied with the relevant provisions of the Chinese national specification “Concrete and Reinforced Concrete Drainage Pipes GB/T 11836” [14].

#### 2.1.3. Design of the Falling Object

**Figure 3:**
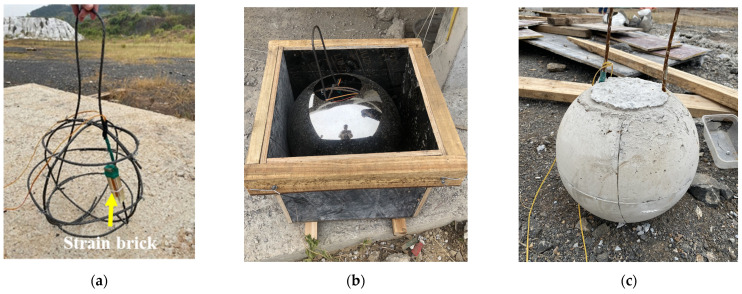
The falling object: (**a**) reinforcing steel bars and strain brick fixed to them; (**b**) spherical mold; (**c**) finished spherical reinforced concrete components.

In an impact experiment, a weight is released from a predetermined height, after which it undergoes the free-fall motion, and the behavior of the weight may change during the falling motion due to air resistance or other forces. In order to control the effect of the falling object’s landing behavior on the test results, spherical reinforced concrete components were designed to replace the falling object. Stainless steel sheets were used to press a spherical mold, which had a diameter of 33 cm, and 0.8 cm thick reinforcement bars were tied internally, on which strain bricks were fixed in order to test the strain of the falling object at the moment of impact on the ground, as shown in Figure 3. The concrete was cast with a strength of 30 MPa and was demolded after consolidation. The mass of the falling stone was 50 kg.

### 2.2. Operating Procedures

**Figure 4:**
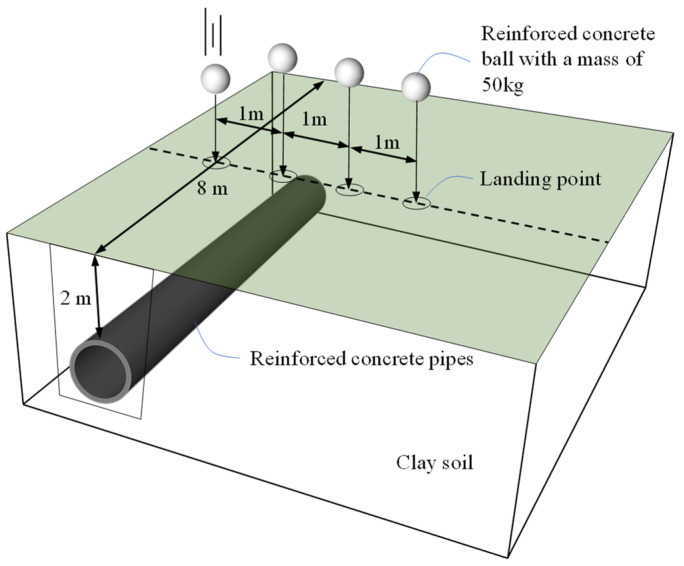
Schematic diagram of the experimental program.

In accordance with the experimental program described above, a general schematic of the test is shown in Figure 4.

**Figure 5:**
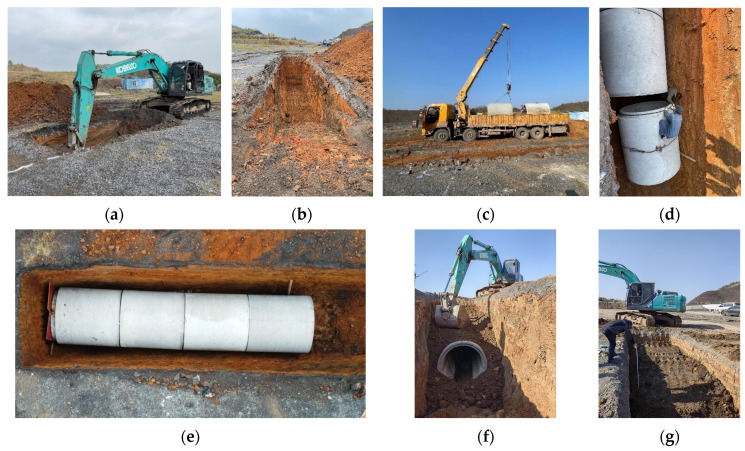
Arrangement of experimental pipes: (**a**) trench excavation; (**b**) pipe trench forming; (**c**) pipe lifting; (**d**) pipe splicing; (**e**) pipe splicing completion; (**f**) backfilling of pipe trenches; (**g**) layered compaction during backfill.

The exact procedure of the experiment was as follows:

Step 1: An excavator was used to dig a pipe trench with a depth of 3.8 m, width of 2 m, and length of 9 m (Figure 5a,b).

Step 2: A crane was used to place the concrete pipes in the pipe trench in sequence and splice them into an 8-meter-long pipeline (Figure 5c–e).

Step 3: After pipeline splicing was completed, the pipe trench was backfilled with the original soil from the site using the excavator. The backfill had to be dense enough to ensure that the soil covering the pipe was in close contact with the pipe (Figure 5f,g).

**Figure 6:**
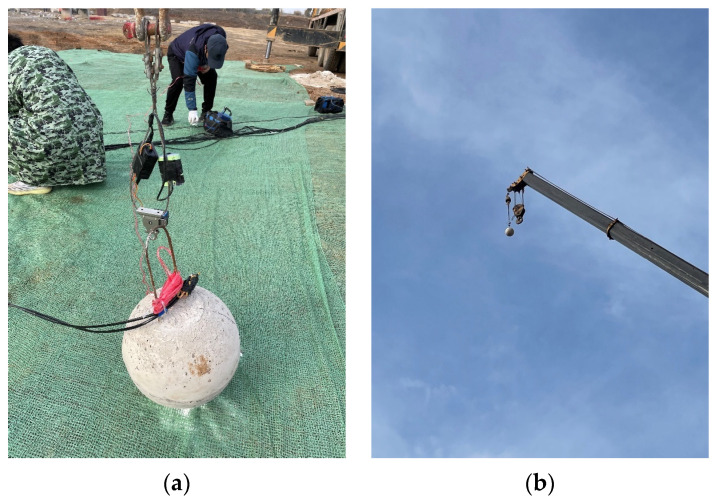
Experimental site: (**a**) decoupler installation; (**b**) concrete ball suspended to a height of 30 m.

Step 4: During the experiment, a remotely controlled decoupler was used to replace the crane’s original hook, and the falling object was lifted to a height of 30 m in the air and kept at that position. After switching on the collection instruments, the test personnel evacuated to a safe position. They then used a remote control to send out a decoupling signal, so that the fixed-point landing ball would land on a predetermined impact point, as shown in Figure 6.

In this section, we reviewed the experimental procedure for characterizing the dynamic response of buried pipelines under surface impact. The tests were carried out at an experimental site with a typical clayey soil layer in the south of Wuhan city. A reinforced concrete pipe was buried in the ground at a depth of 2 m. A concrete ball with a mass of 50 kg was used as a falling object, released from a height of 30 m, and impacted the surface directly above the pipe.

## 3. Dynamic Signal Sensors and Test Methods

### 3.1. Measurement of Dynamic Strain

**Figure 7:**
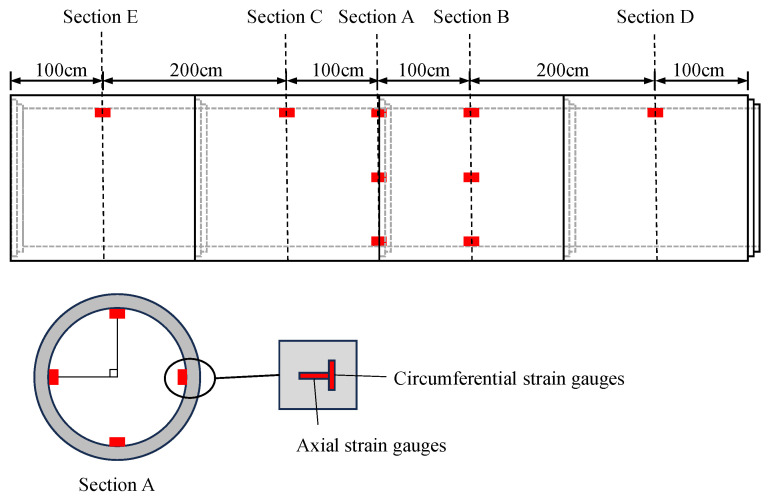
Schematic layout of the strain gauges.

**Figure 8:**
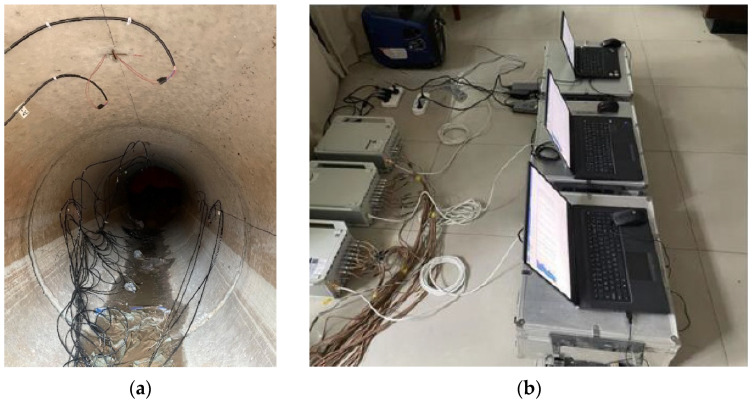
Practical arrangement and connection of strain gauges: (**a**) strain gauges on the inner wall of the pipe; (**b**) DH8302 dynamic signal tester.

Strain gauges were affixed inside the pipe to measure the dynamic strain in the pipe. A total of 5 strain monitoring sections and 11 monitoring points were set up on the inner wall of the pipeline. At each strain measurement point, circumferential and axial strain gauges were affixed separately. Circumferential and axial strain gauges were arranged at the top, waist, and bottom positions of the two sections (Sections A and B). Additionally, in the other three sections (Section C, Section D, and Section E), strain gauges were arranged at the top of the pipe, as shown in Figure 7. The field arrangement of the strain gauges inside the pipe is shown in Figure 8. The length of the strain gauge was 80 mm, and the resistance was 120 Ω. The strain gauges were connected to a dynamic signal test and analysis system using a quarter bridge. The signals collected by the dynamic signal collector were voltage signals, which were converted into strain by setting the gain coefficient. The model number of the dynamic signal collector was DH8302 (Donghua Topology, Taizhou, Jiangsu province, China), with a measurement accuracy of 10 με and a sampling frequency of 2000 Hz.

### 3.2. Measurement of Dynamic Earth Pressure

**Figure 9:**
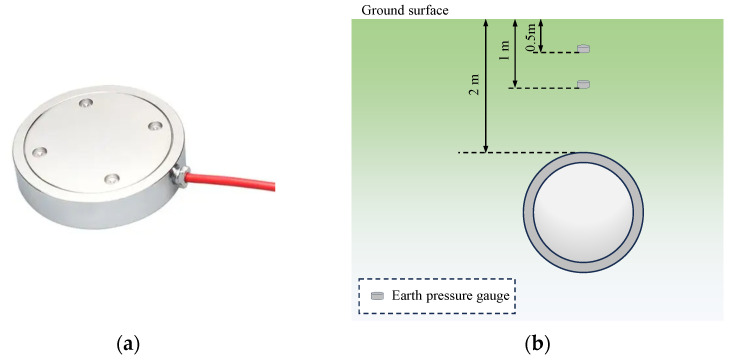
Measurement of earth pressure: (**a**) earth pressure gauge; (**b**) earth pressure gauge measuring point arrangement.

In order to study the characteristics of impact-induced earth pressure changes, in the process of the layered tamped backfilling of in situ soil, earth pressure gauges were deployed directly above the central axis of the pipeline model at 0.5 m and 1 m from the ground surface, as shown in Figure 9. The earth pressure box was connected to the dynamic signal test system using a full bridge, and the collector model was DH8302. The earth pressure gauge at the depth of 0.5 m had a full range of 0.5 MPa, and that at the depth of 1 m had a full range of 0.2 MPa.

### 3.3. Measurement of Vibration Velocity

The velocity of impact-induced vibration is a factor of interest in impact dynamics. Existing studies have shown that the ground vibration velocity is often linked to the dynamic effects of the pipe [15]. Therefore, it is necessary to test the vibration velocity induced by the impact of a falling body on the ground. The MiniMate Plus vibrometer, manufactured by Instantel, Ottawa, Ontario Canada, was used to monitor the vibration velocity in the ground surface induced by the falling object’s impact. The MiniMate Plus vibrometer sensor supports two deployment methods: On hard surfaces such as rock or concrete structures, it can be bonded using quick-setting gypsum, and on soft surfaces such as soils, it can be inserted into the soil using a retainer. Inserting the transducer’s own retainer into the soil connects the vibration sensor to the soil particles to prevent soft soil properties from affecting vibration propagation and monitoring.

**Figure 10:**
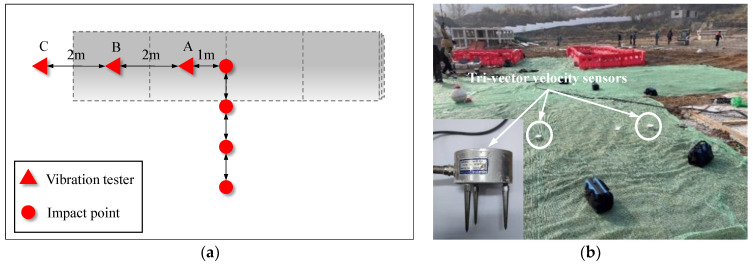
Arrangement of vibration tester: (**a**) schematic diagram of measurement points of vibration tester; (**b**) field arrangement of the vibration tester.

Three vibrometers were deployed along the axis of the pipeline model at 1 m (Point A), 3 m (Point B), and 5 m (Point C) from the impact point, as shown in Figure 10.

### 3.4. Measurement of the Trajectory of Falling Objects

**Figure 11:**
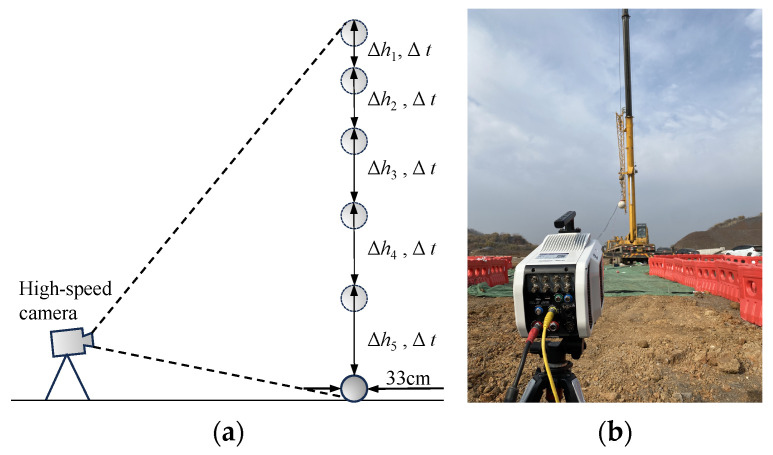
(**a**) Analysis of the velocity of the falling object with a high-speed camera; (**b**) field setup for the high-speed camera.

The velocity of the falling object descending from a height is a significant contributor to the dynamic response of ground and buried pipelines. In order to obtain the falling speed of the falling object, a high-speed camera is used to record the dropping process of the falling object after it is released at a high altitude. Using a high-speed camera ensures that the object under observation has good lighting conditions, and the frequency of the light source is not lower than that of the high-speed camera [16]. The experiment was conducted at noon when the natural light conditions were good, and the frame rate of the high-speed camera was set at 3000 fps. The principle of using a high-speed camera to analyze the velocity of the falling object as well as the field arrangement of the high-speed camera are shown in Figure 11.

In this section, we described the methodology and instrumentation used to observe pipe strains, earth pressure, surface vibration velocities, and falling body velocities during the tests. In the next section, the results obtained in the tests are analyzed.

## 4. Analysis of Experimental Results

### 4.1. Falling Velocity and Pressure of the Falling Object

**Figure 12:**
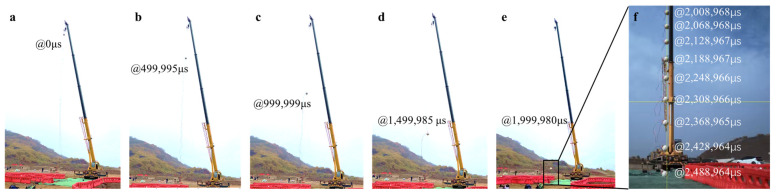
High-speed camera recording the falling of the concrete ball. (**a**) Image at 0 µs; (**b**) Image at 499,995 µs; (**c**) Image at 999,999 µs; (**d**) Image at 1,499,985 µs; (**e**) Image at 1,999,980 µs; (**f**) Image from 2,008,968 µs to 2,488,964 µs.

The observation results of the concrete ball’s landing using the high-speed camera in the first drop experiment were selected and analyzed, and they are shown in Figure 12. The concrete ball lasted a total of 2,488,964 μs in the air, and the trajectory of the ball was extracted using the Revealer Motion Analysis software (V2.1.2); its touchdown velocity was calculated to be about 24.38 m/s.

**Figure 13:**
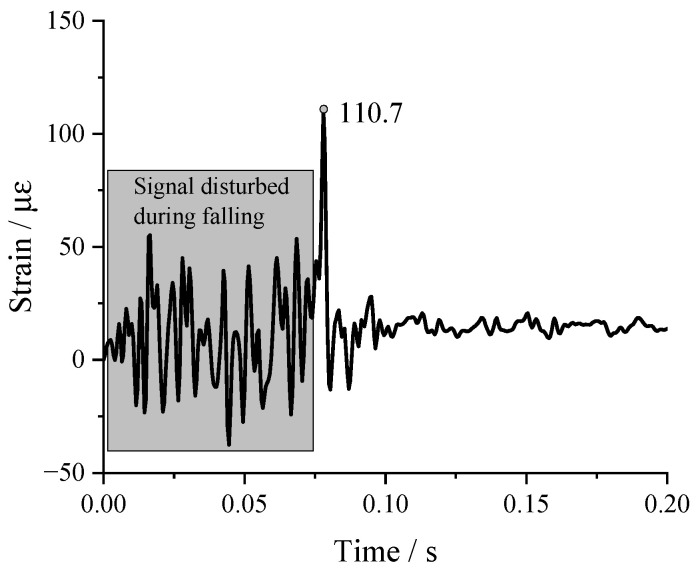
Strain–time history curves inside the concrete ball.

The strain–time curve measured with the internal strain brick during the ball drop is shown in Figure 13. As can be seen from the figure, when the ball was suspended in the air, the ball strain significantly fluctuated, compared with the falling process. At the moment of touchdown, the ball was found to have a strain of 110.7 με. The modulus of elasticity of concrete is about 30 GPa, and using Hooke’s law, the peak stress measured internally when the reinforced concrete ball impacted the ground was calculated, which reached 3.321 MPa. This means that the maximum impact pressure generated by the falling object touching the ground was not less than 3.321 MPa.

**Figure 14:**
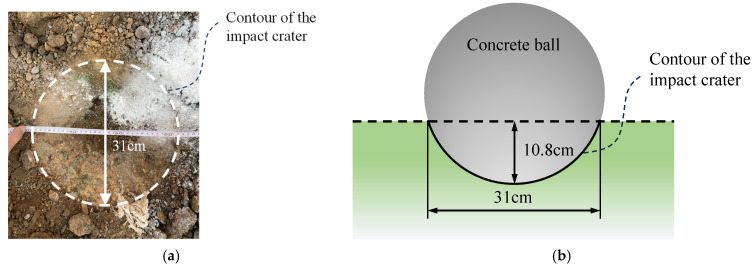
Impact crater: (**a**) impact crater obtained from the experiment; (**b**) schematic dimensions of the impact crater.

As shown in Figure 14, an impact crater with a maximum diameter of about 31 cm and a depth of about 10.8 cm was generated after the ball touched the ground.

### 4.2. Dynamic Pressure in the Soil Induced by Falling Impact

**Figure 15:**
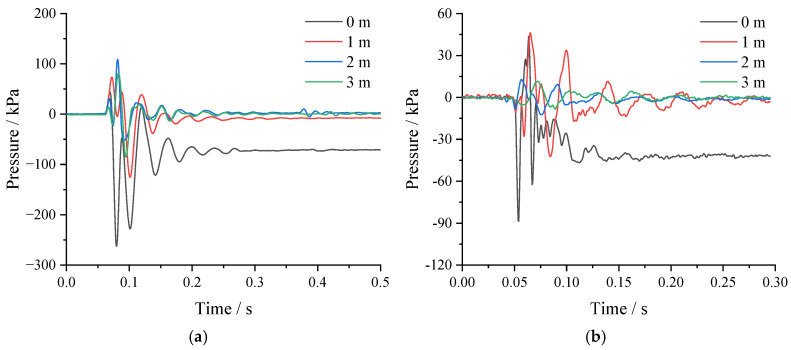
Dynamic earth pressure test results: (**a**) at 0.5 m depth; (**b**) at 1 m depth.

According to the principle of the earth pressure gauge, the earth pressure measured in the test is the product of the strain of the earth pressure gauge and the constant factor K (in kPa). The time curve of the soil pressure under the falling impact, obtained through data processing, is shown in Figure 15. As can be seen from the figure, the peak value of earth pressure at 0.5 m was 259.2 kPa directly below the point of impact. The values measured with the earth pressure gauge at a depth of 0.5 m decreased rapidly as the impact distance increased. The values measured with the earth pressure gauge at a depth of 1 m were relatively small, and their attenuation was relatively small with increasing impact distance. From the test results, it is clear that the impact of a falling object creates permanent additional earth pressure in the soil directly below the point of impact.

### 4.3. Surface Vibration Due to Impact of Falling Impact

**Figure 16:**
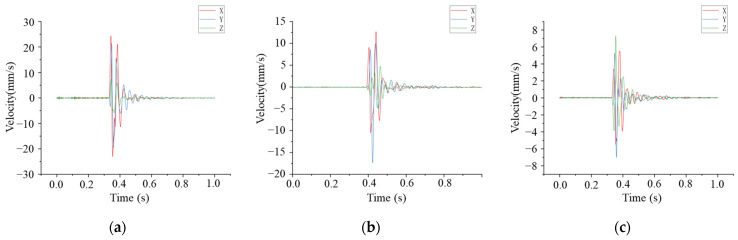
Velocity–time history curve of each measuring point impacting the surface at 0 m: (**a**) Point A; (**b**) Point B; (**c**) Point C.

**Figure 17:**
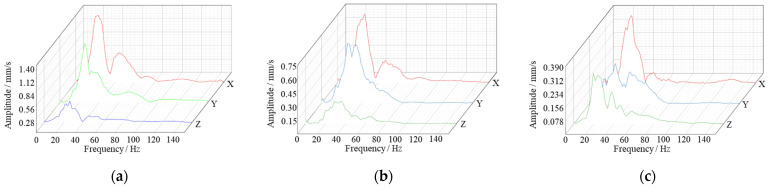
Vibration frequency distribution and amplitude of each measuring point on the impact surface at 0 m: (**a**) Point A; (**b**) Point B; (**c**) Point C.

According to the above test scheme, the vibration monitoring data of the soil surface under the falling impact were obtained, and the vibration velocity and dominant frequency in each direction under different working conditions are shown in Table 3. According to the statistical results in the table, the vibration velocity of the soil surface decreased along the axis direction of the pipeline, and at the same vibration measurement point, the vibration velocity decreased with the increase in the distance of the impact point from the pipeline model. The dominant frequency in each direction was determined. The vibration velocity in the Z direction was smaller than X and Y, while its frequency was larger than X and Y. This is because the impact vibration wave is located on the surface of the soil body, so the surface of the soil body is more susceptible to the influence of the surface wave; thus, the vibration velocity in the horizontal direction is larger than that in the vertical direction. The time course curve of vibration velocity on the surface impacted directly above the pipeline model is shown in Figure 16. The vibration frequency distribution of the maximum vibration measurement point at the surface is shown in Figure 17.

### 4.4. Dynamic Strain in Pipes Due to Falling Impact

#### 4.4.1. Impact Point Located Directly above the Pipe

**Figure 18:**
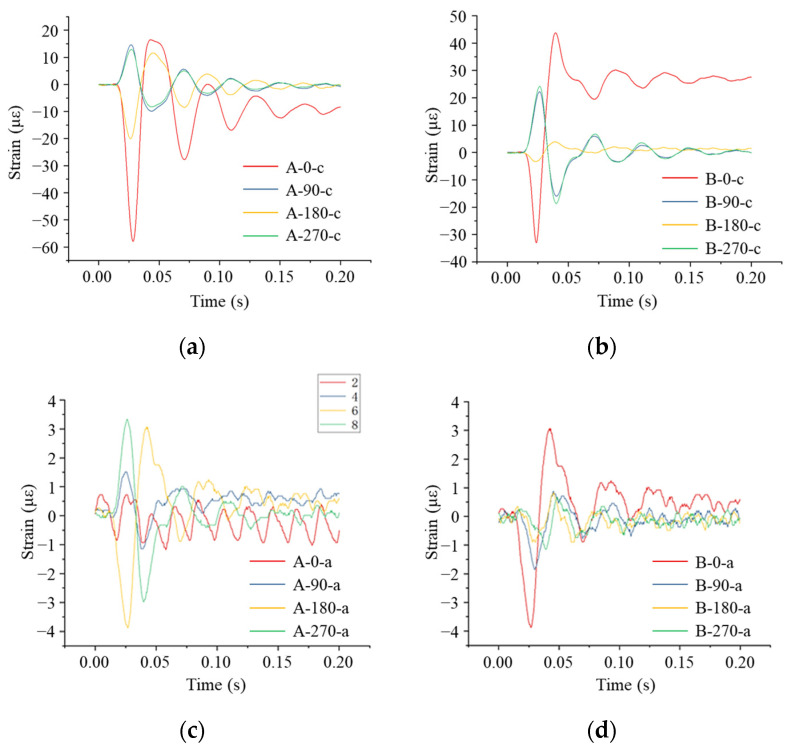
Strain monitoring results of the pipe when the impact point was located directly above the pipe: (**a**) circumferential strain at each monitoring point at Section A; (**b**) circumferential strain at each monitoring point at the B interface; (**c**) axial strain at each monitoring point at the A interface; (**d**) axial strain at each monitoring point at the B interface.

From Figure 18a,b, it can be seen that the response times of the strain measurement points at the same cross-section of the pipe did not differ significantly. Measurement Points A-0-h and B-0-h were located at the top of the pipe model. The A-0-h measurement point was located directly below the impact, and at the A-0-h measurement point, a compressive strain was first generated, which reached a peak value of 57.97 με, and then it began to decrease and shift to the tensile state. The strain variation characteristics of the B-0-h measurement point were basically the same as that of the A-0-h measurement point, which was numerically smaller than that of the A-0-h measurement point, with the maximum compressive strain of 34.28 με and the maximum tensile strain reaching 45.17 με. The A-180-h measurement point was located at the bottom of the pipe model, and the strain change trend was basically the same as that at the top, with the maximum compressive strain of 19.81 με. The trend of strain change at the B-180-h measurement point was basically the same as that at the A-180-h measurement point but numerically smaller. The measurement points A-90-h and A-270-h were located at the waist of the pipe model. First, the tensile strain was generated, and then it began to decrease and turn into compression; the maximum tensile strain was 16.78 με. The strain trends of the two measurement points B-90-h and B-270-h were basically the same as those of the A-90-h and A-270-h measurement points.

From Figure 18c, it can be seen that in the axial direction at the top of the pipe model, the compressive strain was first generated, which reached a maximum of 3.87 με, and then it started to decrease and turn into the tensile strain, which reached a maximum of 2.99 με. In the axial direction of the waist of the pipe model, the tensile strain was first generated, after which it started to decrease and turn into compression. As can be seen in Figure 18d, the axial strain at the top of the pipe model similarly decreased with an increase in the impact distance.

Comparing the circumferential strain and the axial strain, we found that the axial strain of the pipe model was smaller than the circumferential strain, which indicates that the circumferential strain was the dominant strain in the pipe model when subjected to the collapsing impact load, with the axial strain serving as a supplementary force.

#### 4.4.2. Impact Point 1 m away from the Pipe Axis

**Figure 19:**
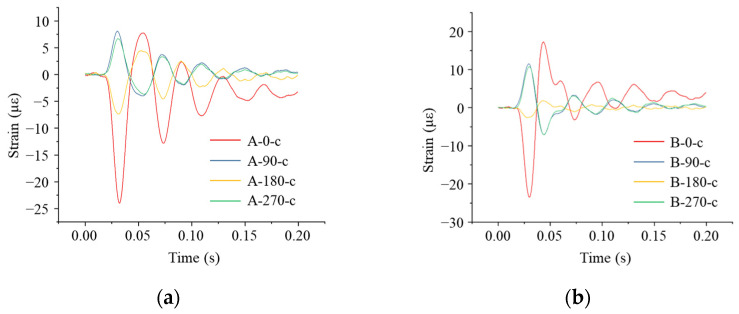
Strain monitoring results of the pipe when the impact point was 1 m away from the pipe axis: (**a**) circumferential strain at each monitoring point in Section A; (**b**) circumferential strain at each monitoring point in Section B.

As can be seen from Figure 19, compared with the strain value of the pipeline obtained when the impact point was located directly above the pipeline model, the strain value was smaller when the impact on the ground occurred at a distance of 1 m from the pipeline, but the change rule of the strain was basically consistent. The maximum compressive strain was observed at the top of the pipe at measurement point A-0-h, with a maximum compressive strain of 24.31 με. The compressive strain at measurement point A-0-h was 22.42 με. In both Sections A and B, the strain at the waist of the pipe was tensile and then changed to compression, and the values of the strains were under 10 με.

#### 4.4.3. Impact Point 2 m away from the Pipe Axis

**Figure 20:**
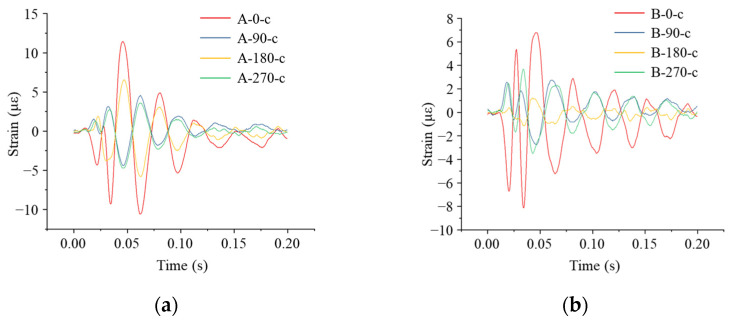
Strain monitoring results of the pipe when the impact point was 2 m away from the pipe axis: (**a**) circumferential strain at each monitoring point in Section A; (**b**) circumferential strain at each monitoring point in Section B.

As shown in Figure 20, the maximum strain occurred at the A-0-h measurement point at the top of the pipe model, and the pipe model was subjected to compression followed by tension, with a maximum tensile strain of 11.43 με and a maximum compressive strain of 10.48 με. It was found that when the impact position changed, the strain inside the pipe underwent some change, and the tensile strain at the top of the pipe was greater than the compressive strain. The strain at the waist of the pipe in both sections was first tensile and then changed to compression, and the strain values were below 5 με.

#### 4.4.4. Impact Point 3 m away from the Pipe Axis

**Figure 21:**
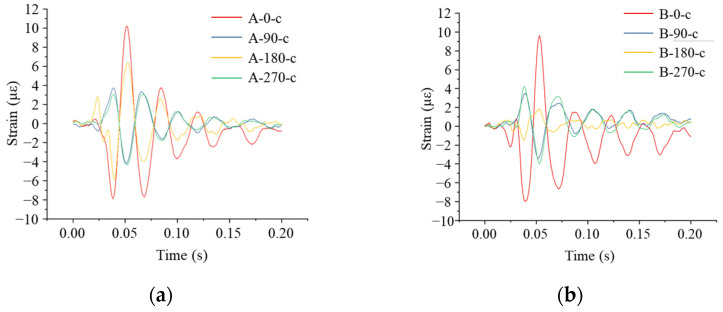
Strain monitoring results of the pipe when the impact point was 3 m away from the pipe axis: (**a**) circumferential strain at each monitoring point in Section A; (**b**) circumferential strain at each monitoring point in Section B.

As shown in Figure 21, the maximum strain occurred at the top of the pipe at the measurement point A-0-h. The pipe was subjected to compression first and then to the tensile strain; the maximum tensile strain was 10.16 με, and the maximum compressive strain was 7.85 με. The tensile strain at the top of the pipe was larger than the compressive strain.

According to the strain characteristics of the pipe for the two cases of the impact points 2 m and 3 m away from the pipe axis, it can be hypothesized that when the impact point is located diagonally above the pipe, the dominant strain at the top of the pipe will be the tensile strain, and the failure mode of the pipe will be tensile damage.

#### 4.4.5. Strain Distribution Law of Pipeline under Falling Impact

**Figure 22:**
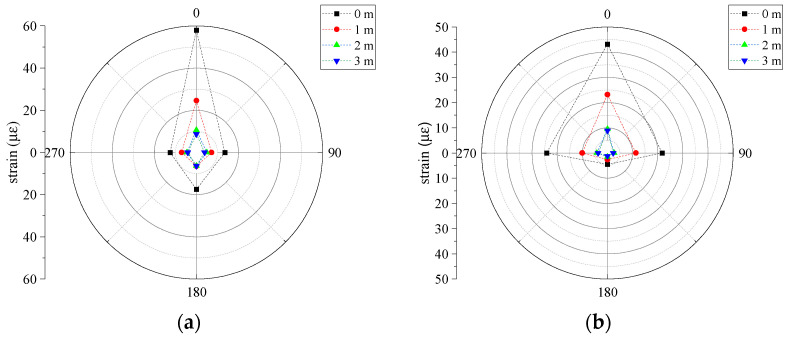
The circumferential strain of each strain monitoring section at different impact points: (**a**) circumferential strain in Section A; (**b**) circumferential strain in Section B.

The circumferential strain distribution of each strain monitoring section at the impact location under different working conditions is shown in Figure 22. It can be seen that the dominant dynamic strain on the cross-section of the pipe model is the annular strain. Additionally, under different working conditions, the strain of the pipe model gradually decreased with the increase in the impact distance, and the annular strain of the pipe model reached the maximum when the impact occurred directly above the pipe.

The pipe showed different strain change characteristics when impacted at different locations. When the impact point was directly above or close to the pipe, the dominant strain generated at the top of the pipe model was the compressive strain, which then changed to a tensile state, but the compressive strain was always greater than the tensile strain. When the impact occurred far away from the pipe, the pipe was first subject to the compressive strain and then the tensile strain, but the resulting tensile strain was larger than the compressive strain. The tensile strain started to become the dominant strain in the pipe, and the failure mode changed to tensile damage.

**Figure 23:**
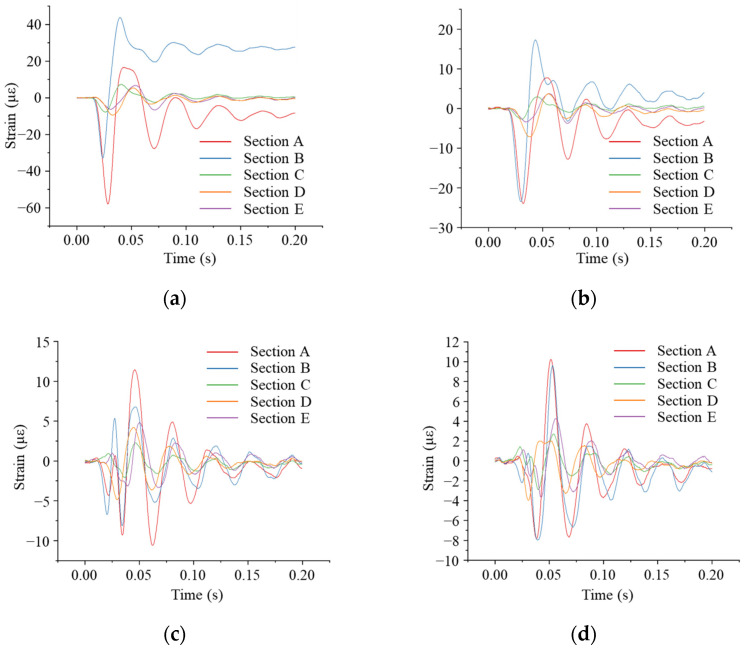
Circumferential strain at tunnel top at different impact points: (**a**) impact point located directly above the pipe; (**b**) impact point 1 m away from the pipe axis; (**c**) impact point 2 m away from the pipe axis; (**d**) impact point 3 m away from the pipe axis.

From Figure 23, it can be seen that the annular strain of the pipe under impact loading varied significantly along the pipe axis. The circumferential strain was greatest at Section A of the pipe (the cross-section closest to the point of impact) and decreased the farther the cross-section was from the point of impact. The cross-section of the pipe closest to the point of impact was most susceptible to damage.

In this section, we analyzed the data obtained from the tests. The results show that the closer the impact distance is, the stronger the dynamic response of the pipe is. The circumferential strain value of the pipe was much larger than the axial strain value. The top of the pipe was the most vulnerable point under surface impact.

## 5. Conclusions

In this study, a prototype test of the dynamic response of a buried pipeline under the action of a falling impact was designed and conducted. Through the use of multiple dynamic signal sensors, the results of surface vibration, soil dynamic pressure, and dynamic strain in the pipeline under the action of falling impact were obtained. Based on the test results, the dynamic response characteristics of buried pipelines under impact loads were analyzed. The main conclusions are as follows:

1. In the experiment, the earth pressure at 0.5 m directly below the impact point of the falling object could reach 259.2 kPa, and the earth pressure at 1 m directly below the impact point was 88.6 kPa. The dynamic pressure of the soil body decreased more rapidly in the soil. From the characteristics of the soil pressure–time curve, we found that the impact of the falling object generates permanent additional pressure in a certain range of soil.
2. The main shock directions of ground vibration under the falling impact load were horizontal and horizontal tangential directions. The vibration caused by the falling impact on the ground attenuation speed was faster. The shock vibration frequency was between 10 Hz and 40 Hz. This frequency is between the natural earthquake and the blasting vibration frequency.
3. Under the impact, the dominant dynamic strain of the buried pipeline was the circumferential strain. When the point of impact was located above the pipeline, the maximum strain of the pipeline was the compressive strain. When the point of impact was located diagonally above the pipeline, the dominant strain at the top of the pipeline model was the tensile strain, and the failure mode of the pipeline was tensile damage. The cross-section of the pipe closest to the impact point was the most susceptible to damage.
4. In this paper, the experimental process of buried reinforced concrete pipelines under the impact of a falling object was described in detail, and the test data obtained were analyzed to provide a preliminary understanding of the dynamic response characteristics of buried pipelines under surface impact. The study results reveal the possible damage modes of the pipeline under the action of ground impact and provide a direction for the design of the impact resistance of buried reinforced concrete pipelines.
5. The experiments conducted in this study have some limitations. The quantity of data obtained in the tests was limited, and the soil around the pipeline inevitably underwent deformation as the test progressed, leading to changes in the initial conditions. In the next step, numerical simulations will be used to more extensively study the dynamic response characteristics of pipelines under the surface impact, and at the same time, other factors such as using a falling object with a different shape, different falling velocities, and different depths of the pipeline and pipe diameters will be taken into account.

## Figures and Tables

**Table 1:** Physical and mechanical parameters of the soil.

No.	Soil Type Name		Measured Values of Geotechnical Experiments		
		Poisson’s Ratio	Density (ρ/cm^3^)	Cohesion	Internal Friction Angle
1	Miscellaneous fill	-	1.80	4	18
2	Silty clay	0.3	1.90	34	13.8

**Table 2:** Detailed parameters of the pipes.

Inner Diameter (m)	Wall Thickness (m)	External Diameter (m)	Concrete Consumption		
			Volumetric (m^3^)	Weight (kg)	
1.5	0.15	1.8	0.777	1865	
Reinforcing steel consumption (kg)	Weight (kg)	Minimum reinforcing area (mm^2^)		External pressure load (kN)	
		Internal layer	Outer layer	Cracks	Destroy
78	1943	707	441	99	150

**Table 3:** Surface monitoring velocity at each point.

Impact Point	Monitoring Point	*v*_X_/m·s^−1^	*f*_X_/Hz	*v*_Y_/m·s^−1^	*f*_Y_/Hz	*v*_Z_/m·s^−1^	*f*_Z_/Hz
0 m	A	2.43	23.2	2.11	26.0	0.74	26.5
	B	1.27	27.0	1.73	27.0	0.47	30.5
	C	0.55	26.2	0.70	26.8	0.72	20.5
1 m	A	2.23	22.2	2.01	26.8	0.46	27.8
	B	0.98	28.2	1.44	36.0	0.42	40.2
	C	0.44	27.2	0.49	27.0	0.59	30.8
2 m	A	1.54	25.0	1.37	26.6	0.38	27.5
	B	0.59	28.5	1.34	28.0	0.44	34.2
	C	0.21	25.8	0.15	27.4	0.29	26.6

## Data Availability

Data are contained within the article.
